# Pharmaceutical Biotechnology

**DOI:** 10.3390/life12081240

**Published:** 2022-08-16

**Authors:** Cesar Augusto Roque-Borda, Fernando Rogério Pavan, Andréía Bagliotti Meneguin

**Affiliations:** School of Pharmaceutical Sciences, São Paulo State University (UNESP), Araraquara 14800-903, SP, Brazil

Biotechnology and pharmacy have shown efficient results when combined to generate innovative technological products [1]. In recent years, the increase in infectious diseases has led to economic decline and a global crisis [2]. The COVID-19 pandemic produced by SARS-CoV-2, a type of coronavirus, has taken the lives of millions of people in the last two years [3]. During this waiting time, the treatments adopted for infected patients have been based on the administration of azithromycin, and the effects of the uncontrolled use of this medication will be seen in years to come [4].

Pharmaceutical biotechnology has made it possible to quickly obtain vaccines and new recombinant drugs or drugs derived from living organisms that have a broad spectrum or specific action and manage to prevent diseases [5]. However, when it comes to infectious diseases, obtaining new drugs approved by the FDA is difficult because the ratio between reports of new MDR strains and new drugs is significantly large [6]. Concerning *Mycobacterium tuberculosis*, the bacterium that claims the most lives in the world, the registration of drugs is scarce or virtually non-existent [7]. The last drug to be approved (in 2012) was bedaquiline, and the bacterium has already shown resistance to almost all the drugs on the market. The seriousness of this problem led to a bedaquiline–protomanid combination (synergistic effect) to be approved in 2019 [8]. Therefore, the urgency for obtaining new drugs, especially for MDR bacteria classified as “critical and high priority” by the WHO, should be highlighted [9].

Thus, we have launched this Special Edition of Life called *Pharmaceutical Biotechnology*, where we frame the use of biomacromolecules that are being studied in depth, such as antimicrobial peptides, lipids, metal complexes, proteins, and toxins. Some molecules such as *N*-palmitoylethanolamide-oxazoline, i.e., an endogenous lipid, are able to regulate homeostasis and are involved in the modulation of inflammation; consequently, this molecules were shown to reduce intestinal damage in an animal model, namely zebrafish larvae [10]. This is an animal model with a high impact because it enables the replacement or reduction in the use of experimental mice [11].

Moreover, nanotechnology has shown promising results in the administration of drugs and the improvement of their activity. In this Special Issue, Salem et al. [12] report a study on selenium nanoparticles obtained from orange peel waste. These authors studied their properties against multidrug-resistant (MDR) bacteria such as *P. aeruginosa, MDR E. coli*, *K. pneumonia*, *S. aureus* ATCC 29213, and MDR clinical isolates, as well as their potential against the formation of biofilm of *S. aureus* clinical isolates. It is worth noting that these nanoparticles were obtained through green and environmentally friendly procedures, which is crucial in these times of environmental crisis [13]. Furthermore, a controlled transport mechanism in liquid crystals reported by Santos et al. [14] demonstrates the ability to sublingually administer rifampicin, with dose control and excellent anti-*Mycobacterium tuberculosis* activity after seven days of treatment.

Another highlight of these nanoformulations is reported by Elsewedy et al. [15]. These authors developed a hydrogel based on tea tree oil nanoemulsions for the purpose of improving the topical administration of neomycin, a drug used for bacterial skin infections. They demonstrate that the formulation was stable for up to three months and that there was no evidence of skin irritation. Likewise, Di Filippo et al. [16] report the development of microparticles formulated on the basis of chitosan loaded with ascorbic acid and nicotinamide, showing good physicochemical stability and dose-regulated protective antimicrobial activity. The authors demonstrated that there was no ex vivo permeation of the pig ear, which is excellent for the development of cosmetic formulations for the skin.

In summary, these results allow us to appreciate the great opportunities that the use of nanotechnology offers in the fight against MDR bacteria, improving the stability and administration of drugs against diseases or metabolic deregulations [5].

## Data Availability

Not applicable.
